# Metabolic targeting of multiple myeloma using the Warburg Trap

**DOI:** 10.1186/s12935-026-04504-y

**Published:** 2026-11-13

**Authors:** Nina Hildenbrand, Elena Tripel, Svitlana Melnik, Daniel Strelzov, Alexandra M. Poos, Lukas John, Niels Weinhold, Marc A. Baertsch, Tilman Walker, Burkhard Lehner, Jörg Fellenberg

**Affiliations:** 1 Research Centre for Molecular and Regenerative Orthopaedics, Department of Orthopaedics, Heidelberg University Hospital, Heidelberg, Germany; 2 Department of Orthopaedics, Medical Faculty, Heidelberg University Hospital, Heidelberg University, Heidelberg, Germany; 3 Heidelberg Myeloma Center, Department of Internal Medicine V, Medical Faculty, Heidelberg University Hospital, Heidelberg University, Heidelberg, Germany; 4 Clinical Cooperation Unit Molecular Hematology/Oncology, Department of Internal Medicine V, Heidelberg University Hospital, Heidelberg University, German Cancer Research Center (DKFZ), Heidelberg, Germany; 5 Department of Orthopaedics, Heidelberg University Hospital, Schlierbacher Landstrasse 200a, D-69118 Heidelberg, Germany

**Keywords:** Multiple myeloma, Cancer metabolism, Warburg effect, Warburg Trap, Metabolic cancer therapy, Metformin

## Abstract

**Background:**

The Warburg effect, characterized by enhanced glycolysis and lactate production, is a universal feature of cancer cells, even under normoxic conditions. The resulting extracellular acidification and intracellular alkalization create a selective vulnerability that is absent in normal tissues. We previously developed the Warburg Trap (WT) approach, which exploits this vulnerability by combining inhibitors of mitochondrial complex I with H^+^-ionophores to collapse pH homeostasis and energy balance, thereby inducing cancer-specific cell death. Given that the bone marrow microenvironment in multiple myeloma (MM) is markedly acidic (pH 6.7–6.8 compared to ~ 7.4 in normal bone marrow), we hypothesized that MM cells would be highly sensitive to WT treatment.

**Methods:**

The effects of individual WT drugs and drug combinations on three human myeloma cell lines (hMCLs), primary MM cells and one non-neoplastic human osteoblast-like cell line (hOBs) were analyzed in vitro. To evaluate the cytotoxic effects, the IC_50_ values of the drugs were calculated, and the Zero Interaction Potency (ZIP) model was used to identify possible synergies. Cell viability, intracellular pH values (pHi), the invasive potential, apoptosis induction, glucose uptake, ATP levels, mitochondrial depolarization and glucose transporter expression were analyzed using fluorescence microscopy, flow cytometry, RT-qPCR analysis and various in vitro assays.

**Results:**

Combinations of the mitochondrial complex I inhibitors metformin (Me) and fenofibrate (Fe) with one of the H^+^-ionophores drotaverine (Dro) or monensin (Mo) significantly induced apoptosis in hMCLs and primary MM cells, while hOBs remained unaffected. The triple medication regimen exhibited a clear synergistic effect compared to monotherapy. Mechanistic analyses revealed a pronounced drop in pH_i_, disruption of glucose metabolism, depletion of ATP and loss of mitochondrial potential in response to these drugs. Furthermore, the WT drugs suppressed the invasive potential of MM cells and induced the upregulation of the stress-associated gene DDIT3 and the encoded CHOP protein. RT-qPCR profiling of solute carrier family 2 A (*SLC2A*) genes, encoding glucose transporters (GLUTs) revealed that *SLC2A1*,* SLC2A8* and *SLC2A9* were the most abundant transcripts in MM cells, with *SLC2A8* showing the most pronounced difference between MM cells and non-neoplastic hOBs. Additionally, *SLC2A8* expression associated with the observed WT-drug sensitivity of the analyzed cell lines.

**Supplementary Information:**

The online version contains supplementary material available at https://doi.org/10.1186/s12935-026-04504-y.

## Introduction

Multiple myeloma (MM) is a malignant hematological disease that originates in the bone marrow. It is characterized by the clonal proliferation of malignant plasma cells and the presence of monoclonal proteins in serum and/or urine [1]. Current standard of care includes induction therapy with a combination of an anti-CD38 monoclonal antibody (e.g. daratumumab, isatuximab), proteasome inhibitors (e.g. bortezomib), lenalidomide and dexamethasone, followed by high-dose chemotherapy, autologous stem cell transplantation and maintenance therapy [2, 3]. For relapsed or refractory disease, advanced therapeutic therapies such as chimeric antigen receptor (CAR) T-cell therapies (e.g. ciltacabtagene autoleucel) and bispecific antibodies (e.g. teclistamab) have further expanded the treatment landscape [4, 5]. However, despite therapeutic advances, MM remains incurable, with a 5-year relative survival rate of ~ 62% and mortality approaching 117,000 deaths globally each year [6]. This underscores the urgent need for novel, clinically translatable strategies to improve outcomes in MM.

Recent studies have indicated distinct metabolic alterations in MM cells, including enhanced glutaminolysis, lipogenesis, and altered mitochondrial dynamics alongside heightened glucose dependence and a preference for aerobic glycolysis over oxidative phosphorylation, even when oxygen is present, a phenomenon known as the Warburg effect [7–10]. Upregulation of key glycolytic enzymes, such as hexokinase II (HK2) and pyruvate kinase M2 (PKM2), facilitates the conversion of glucose to lactate even under normoxic conditions, directly driving this Warburg phenotype [9, 11]. To maintain high glycolytic flux and prevent cellular acidosis, MM cells overexpress proton-linked monocarboxylate transporters (MCT1/4) alongside the chaperone protein CD147, which facilitates lactate export [9, 11]. Elevated expression of these glycolytic regulators directly correlates with chemoresistance and poorer overall patient survival [9]. Presumably, the accumulation of exported lactate likely serves as a contributing factor to the altered extracellular pH observed within the MM bone marrow microenvironment.

Compared to normal bone marrow with an extracellular pH of approximately 7.4, comparable to that of blood, the bone marrow microenvironment in MM exhibits a markedly acidic shift [12]. In vivo measurements using AcidoCEST MRI revealed that the extracellular pH in MM ranges between 6.7 and 6.8 [12]. This substantial deviation from physiological pH reflects the metabolic reprogramming characteristic of malignant plasma cells and creates a unique biochemical niche within the marrow. Such acidity not only influences tumor progression and drug resistance but also presents a selective vulnerability that might be exploited therapeutically.

Additionally, the elevated glucose uptake in MM has already been utilized as a valuable clinical tool via fluorodeoxyglucose positron emission tomography (FDG-PET). Both elevated glucose uptake by MM cells and the number of FDG-PET-positive lesions at initial diagnosis serve as strong prognostic indicators for MM patients [13].

Collectively, the pronounced acidity of the MM microenvironment, high glucose uptake, and hallmark reliance on the Warburg effect provide a compelling rationale for a Warburg effect–targeted therapy, offering an ideal framework to exploit this therapeutic strategy in MM.

We have previously developed the Warburg Trap (WT) approach, which specifically exploits the Warburg effect in cancer cells by combining mitochondrial complex I (MCI) inhibitors (e.g., metformin, fenofibrate) with H^+^-ionophores (e.g., drotaverine, monensin) [14, 15]. Mechanistically, H^+^-ionophores act as lipophilic proton shuttles that uncouple the transmembrane proton gradient, causing severe intracellular acidification, mitochondrial membrane potential collapse, and ATP depletion to selectively trigger cancer-specific cell death [14, 15]. We have previously shown the selective cytotoxicity of this approach on osteosarcoma cell lines, where it induced intracellular acidification and apoptotic cell death, whereas non-neoplastic human osteoblasts tolerated the treatment [14].

To transfer this approach from primary bone tumors to a primary bone marrow malignancy such as MM, herein, we evaluated the first in vitro approach of our previously described drug combination of MCI inhibitors with H^+^-ionophores on MM cell lines. In our in vitro models, these WT-drug combinations induced a strong synergistic effect leading to a reduced viability of MM cell lines, whereas osteoblasts appeared largely unaffected under the tested conditions. Preceding the observed cell death, we detected a significant drop of intracellular pH-values (pHi), accompanied by a massive increase in glucose uptake and a rapid depletion of ATP levels in MM cells. These data support our hypothesis of a possible MM treatment strategy by targeting the Warburg effect.

Furthermore, variations in treatment sensitivity were observed in relation to GLUT expression patterns, suggesting a potential association between *SLC2A8* expression levels and susceptibility to Warburg-targeted interventions. Overall, these preliminary in vitro findings suggest that the WT approach may offer a viable strategy for targeting metabolic vulnerabilities in MM, warranting further investigation in preclinical in vivo models.

## Methods

### MM cell lines and primary human osteoblasts

The commercially available human myeloma cell lines (hMCLs) MM1.S, OPM-2 and RPMI-8226 were kindly provided by the Heidelberg Myeloma Center, Department of Internal Medicine V, Heidelberg University Hospital and German Cancer Research Center (DKFZ), Heidelberg, Germany. Cells were cultured for the first week after thawing in RPMI-1640 medium (Thermo Fisher Scientific, Dreieich, Germany) containing 20% fetal bovine serum (FBS; Bio&SELL GmbH, Feucht, Germany, lot number BS.371356.5D) and 100 U/mL penicillin/streptomycin (Sigma-Aldrich) for expansion. Cultivation was subsequently carried out in RPMI-1640 medium with 10% FBS and 100 U/mL penicillin/streptomycin, with medium changes three times per week.

Human osteoblasts (hOBs) were isolated (as approved by the local ethics committee: S-086/2023) at the Department of Orthopaedics, University Hospital Heidelberg, Germany, from patients undergoing total knee arthroplasty. Bone samples were cut into small fragments and washed several times with phosphate buffered saline PBS (Thermo Fisher Scientific). Tissue digestion of the bone fragments was performed using Dulbecco’s Modified Eagle Medium (DMEM) with 1 mg/mL of collagenase type II (Thermo Fisher Scientific) for 2 h at 37 °C under continuous rotation. Following digestion, the cells were washed twice with PBS and subsequently cultured in DMEM, which contained 1 g/L glucose, 10% FBS, 100 U/mL penicillin/streptomycin, and 0.17 mM ascorbic acid 2-phosphate (Sigma-Aldrich). After a 24-hour period of incubation, non-adherent cells were removed without compromising the integrity of the bone fragments. At 80% confluency, the cells were passaged, and the bone fragments were discarded.

### Primary human MM cells

Primary patient sample was contained at the Department of Orthopaedics, Heidelberg University Hospital, Germany, after written informed consent for the use in research and analysis was obtained (as approved by the local ethics committee, license number: S-369/2024). The primary MM sample (*n* = 1) was collected from a MM lesion in the proximal femur of a patient undergoing medically indicated orthopedic surgery. Fresh tumor tissue was immediately transferred to the laboratory and minced into small pieces. Cell isolation was performed through tissue digestion in an enzyme mix, consisting of 8 mg Dispase (Thermo Fisher Scientific, Lot 2581594), 10 mg Collagenase-II (Thermo Fisher Scientific, Lot 2850061) and 1 mg DNAse (Roche Diagnostics, Lot 80781100) in 10 ml RPMI medium (Thermo Fisher Scientific, Lot 3062437). Incubation was performed at 37 °C temperature for 15 min. The sample was then filtered through a 100 μm cell strainer with Quenching Puffer (5 ml 0.5 M EDTA and 10 ml FBS in 485 ml PBS) and centrifuged. Red blood cell lysis was performed on ice for 3 min using a 1:10 dilution of 10x ACK lysis buffer (43 g of 1.5 M NH₄Cl, 5 g of 100 mM KHCO₃, and 1 mL of 0.5 M EDTA in 500 mL of Milli-Q water). Centrifugation at 460 g for 5 min was performed between each step to collect and resuspend the cell pellet. After red blood cell lysis, the cell pellet was washed and resuspended in PBS and filtered through a 100 μm cell strainer. The cells were counted and resuspended in a freezing medium consisting of 90% FBS and 10% sterile dimethyl sulfoxide (DMSO; Roth). Cells were transferred to cryotubes and cryopreserved in liquid nitrogen until analysis.

Cells were cultured for the first week after thawing in RPMI-1640 medium containing 20% FBS and 100 U/mL penicillin/streptomycin for expansion. During passaging, adherent cells were removed to isolate the non-adherent cells for further cultivation. The cells were subsequently cultivated in RPMI-1640 medium containing 10% FBS and 100 U/mL penicillin/streptomycin, and the medium was changed three times per week.

### Analysis of cellular viability

Cells were cultivated in a 96-well plate at a density of 15,000 cells per well and treated with either metformin (2 mM), monensin (5 µM), fenofibrate (10 µM), or drotaverine (5 µM) (all from Biomol, Hamburg, Germany), or with a drug combination. The drug combination was used at the indicated concentrations, unless otherwise stated. Treatment was performed for either 24, 48–72 h. For the analysis of cell viability, cells were cultured and treated in round-bottom 96-well plates before propidium iodide (PI) (Miltenyi Biotec) was added to a final concentration of 0.5 µg/ml. Total cell number and the amount of PI-positive, dead cells were quantified in a MACSQuant flow cytometer (MACSQuant Analyzer, MACSQuantifyTM software version 2.13.2, Miltenyi Biotec). The percentage of dead cells was calculated. All experiments were done at least in technical triplicates.

### NucView assay

The induction of apoptosis was quantified by NucView 488 (Biomol) staining, which detects the activation of caspase-3, a hallmark of apoptosis. The cells were seeded at a density of 15,000 cells per well in a black 96-well cell culture plate with a clear bottom. Following this, WT drugs were added at the following concentrations: metformin (2 mM), monensin (5 nM), fenofibrate (10 µM), and drotaverine (5 µM). The apoptosis inducer, staurosporine, was utilized as a positive control, at a concentration of 1 µM. The inhibition of apoptosis was achieved by utilizing the pan-caspase inhibitor zVAD at a concentration of 25 µM. Following a 24-hour or 72-hour treatment period, the cells were subjected to staining with NucView 488 (2.5 µM) diluted in Hank’s balanced salt solution (HBSS) (Sigma-Aldrich) for a duration of 30 min at a temperature of 37 °C. Photographic documentation was conducted using a BZ-X800 microscope (Keyence, Neu-Isenburg, Germany). The quantification of apoptotic cells was conducted by means of flow cytometric analysis of NucView 488-stained cells, utilizing a MACSQuant analyzer. All experiments were done at least in technical triplicates.

### Intracellular pH analysis

Cells were plated in 96-well round-bottom plates at a density of 15,000 cells per well and cultured for 24 h at 37 °C and 5% CO₂ in a humidified atmosphere. After treatment, the medium was replaced by 100 µl staining solution consisting of the pH-sensitive fluorescent dye BCFL (5 µM) (Biomol) diluted in HBSS. Following 30 min incubation at 37 °C, the staining solution was removed, cells were washed twice with HBSS buffer, and fluorescence was quantified at 535 nm using a FluoStar fluorescence microplate reader (BMG LabTech, Ortenberg, Germany). For quantification, standard curves were generated using cells cultured in media with pH-values ranging from pH 6.0 to pH 8.0. By adding the ionophore Nigericin (10 µM) (Sigma-Aldrich), the intracellular pH-value was adjusted to match the extracellular pH-value. This made it possible to generate a standard curve using known pH values. Based on these standards, pH-values of the experimental groups could be estimated. All experiments were done at least in technical triplicates.

### Cell invasion assay

To analyze cellular invasion capacity, the upper surface of ThinCert™ Transwell inserts (Greiner Bio-One) with 8 μm pores was precoated with 50 µL of 1 mg/mL collagen type I (rat tail; ibidi GmbH, Gräfeling, Germany) and incubated overnight at 37 °C and 5% CO₂. Prior to cell seeding, the residual collagen was removed. RPMI-1640 medium containing 10% was added to the lower chamber. hMCLs were serum-starved for 24 h in serum-free RPMI-1640 medium. Then, 300,000 cells were added to the upper chamber of the inserts in a 24-well format and resuspended in one of the following: (i) serum-free medium; (ii) serum-free medium containing a triple-drug combination of metformin (2 mM), drotaverine (5 µM), and fenofibrate (10 µM); (iii) serum-free medium containing 250 ng/ml recombinant CHO-derived Wnt5a (rWnt5a; R&D Systems, Inc., Minneapolis), or (iv) serum-free medium containing both rWnt5a and a triple-drug combination of metformin, drotaverine, and fenofibrate. The inserts were placed in the wells and incubated for 24 h at 37 °C and 5% CO₂. After incubation, the inserts and the medium in the upper chamber were removed, and the cell suspension in the lower chamber was collected. To collect cells that may have attached to the lower surface of the inserts and the wells themselves, the wells were washed with DPBS ( Thermo Fisher Scientific), the lower surface was treated with trypsin (Bio&SELL) for 3 min, and the collected cells were combined with the cell suspension from the lower chamber. The cells were then centrifuged at 200 g for 3 min, resuspended, and quantified utilizing a MACSQuant analyzer. Data were normalized to the untreated serum-free control group. All experiments were done at least in technical triplicates.

### Glucose uptake assay

Glucose uptake of myeloma cells in response to WT drug treatment was quantified using the Glucose uptake-Glo assay from Promega (Promega, Walldorf, Germany) according to the manufacturer’s protocol. In brief, cells were plated in 96-well round-bottom plates at a density of 30,000 cells/well and cultured for 24 h at 37 °C and 5% CO₂ in a humidified atmosphere. As controls, primary hOBs were used at a density of 10,000 cells/well. After WT-drug treatment, cells were washed with PBS before 50 µl of 2-deoxyglucose were added. After a 10 min incubation, the uptake of 2-deoxyglucose was quantified in a luminometer (BMG LabTech) after addition of a specific detection reagent. Glucose uptake was then quantified using a standard curve and expressed as a percentage of untreated control cells. All experiments were done at least in technical triplicates.

### Quantification of ATP

In order to quantify the intracellular ATP levels, the CellTiter-Glo assay was used (Promega), a cell viability assay based on the quantification of ATP. Cells were plated in 96-well round-bottom plates at a density of 20,000 cells/well and cultured for 24 h at 37 °C and 5% CO₂ in a humidified atmosphere. As controls, primary hOBs were used at a density of 10,000 cells/well. After WT-drug treatment, the CellTiter-Glo detection reagent was added to the culture, and the resulting luminescence was quantified after 10 min incubation in a luminometer (BMG LabTech). ATP levels were calculated as a percentage of untreated control cells. All experiments were done at least in triplicates.

### Analysis of mitochondrial membrane potential

For the analysis of the mitochondrial membrane potential, cells were stained with JC-1. In healthy cells this dye forms aggregates that emit red light, while a drop of the mitochondrial membrane potential in unhealthy cells keeps the dye as monomers that emit green fluorescence. Cells were cultured at a density of 50,000 cells/well in 8-well chamber slides (Ibidi GmbH). After the desired treatment, cells were incubated in culture medium supplemented with 1 µM JC-1 (Sigma-Aldrich) for 30 min at 37 °C before cells were washed twice in HBSS and analyzed using a fluorescence microscope (Keyence). Three images were taken at 100x magnification per sample before the areas of red (JC-1-aggregates) and green (JC-1 monomer) fluorescence were quantified using ImageJ software version 1.54 (http://imagej.net/ij/). The ratio of red to green fluorescence was then calculated and used as a relative measurement of mitochondrial potential. Additional images with 600x magnification were taken for better visibility.

### RNA extraction, cDNA synthesis and RT-qPCR

Expression analysis of solute carrier family 2 (SLC2) genes (*SLC2A1*,* SLC2A4*,* SLC2A8* and *SLC2A9*), encoding glucose transporters, and DNA Damage Inducible Transcript 3 (*DDIT3*) was performed by Reverse Transcription quantitative Polymerase Chain Reaction (RT-qPCR). *RPL13* was analyzed as reference gene. For the analysis of hMCLs and primary osteoblasts, cultured cells were used, either untreated or after treatment, as indicated.

RNA isolation was performed by using TRIzol™ (Thermo Fisher Scientific) according to the manufacturer’s protocol. Briefly, the cells were lysed in 1 mL of TRIzol, and then 200 µL of chloroform (Sigma-Aldrich) was added. After shaking and centrifugation, the aqueous phase of the resulting supernatant was collected and precipitated using isopropanol (Carl Roth). The RNA pellet was then washed twice with 70% ethanol, air-dried, and resuspended in 10 mM Tris-HCl (Merck Millipore). RNA quality was controlled using a Spectrophotometer (NanoDrop™, Thermo Fisher Scientific). For cDNA synthesis, 500 ng of RNA was reversely transcribed using Oligo(dT)18 Primer Mix (Fisher Scientific GmbH, Germany) and cDNA quality was then again controlled by using the Spectrophotometer. RT-qPCR was performed using SYBR Green Master Mix (Absolute qPCR SYBR Green Mix, Thermo Fisher Scientific). Primers are listed in Table 1.

**Table 1:** Primers used for RT-qPCR

Gene	Forward primer (5´- 3´)	Reverse primer (5´- 3´)
***SLC2A1***	TTGGCTCCGGTATCGTCAAC	GATGGCCACGATGCTCAGAT
***SLC2A4***	CGTCGGGCTTCCAACAGATA	CCCAGCCACGTCTCATTGTA
***SLC2A8***	CTGGTTCGGGGCTGTCG	ACTGCTGGGTAGGCGATTTC
***SLC2A9***	GTTGGGCTCCTCTTCCCATT	ACGTGGAGGAGGAAACTTGT
***DDIT3***	CAGAGCTGGAACCTGAGGAG	TGGATCAGTCTGGAAAAGCA
***RPL13***	CATTTCTGGCAATTTCTACAG	CAG GCA ACG CAT GAG GAA T

### Immunocytochemistry

Fluorescent immunocytochemistry was performed in round-bottom well plates containing 100,000 cells per well. Following treatment with the desired drugs or drug-combinations, the cells were fixed and permeabilized using ice-cold methanol for 15 min at -20 °C. Following three washes with PBS, the cells were blocked with PBS supplemented with 5% BSA and 0.3% Triton X-100 (Sigma-Aldrich) for one hour at 20 °C. The cells were then incubated overnight at 4 °C with primary anti-CHOP antibody (Cell Signaling, Leiden, The Netherlands), diluted 1:400 in PBS supplemented with 1% BSA and 0.3% Triton X-100. After three further washes in PBS, the cells were incubated with a secondary anti-mouse antibody labelled with AlexaFluor 488 (Cell Signaling), diluted 1:1000, for two hours at 20 °C. The cells were then counterstained with the nuclear dye Hoechst 33,342 (0.25 µg/ml) for 10 min, after which they were washed three times in PBS. The cells were then transferred to chamber slides (ibidi GmbH) and centrifuged at 450 g for 5 min to collect them at the bottom of the wells. Images were taken using a BZ-X800 microscope (Keyence, Neu-Isenburg, Germany).

### Statistics

**Fig. 1:**
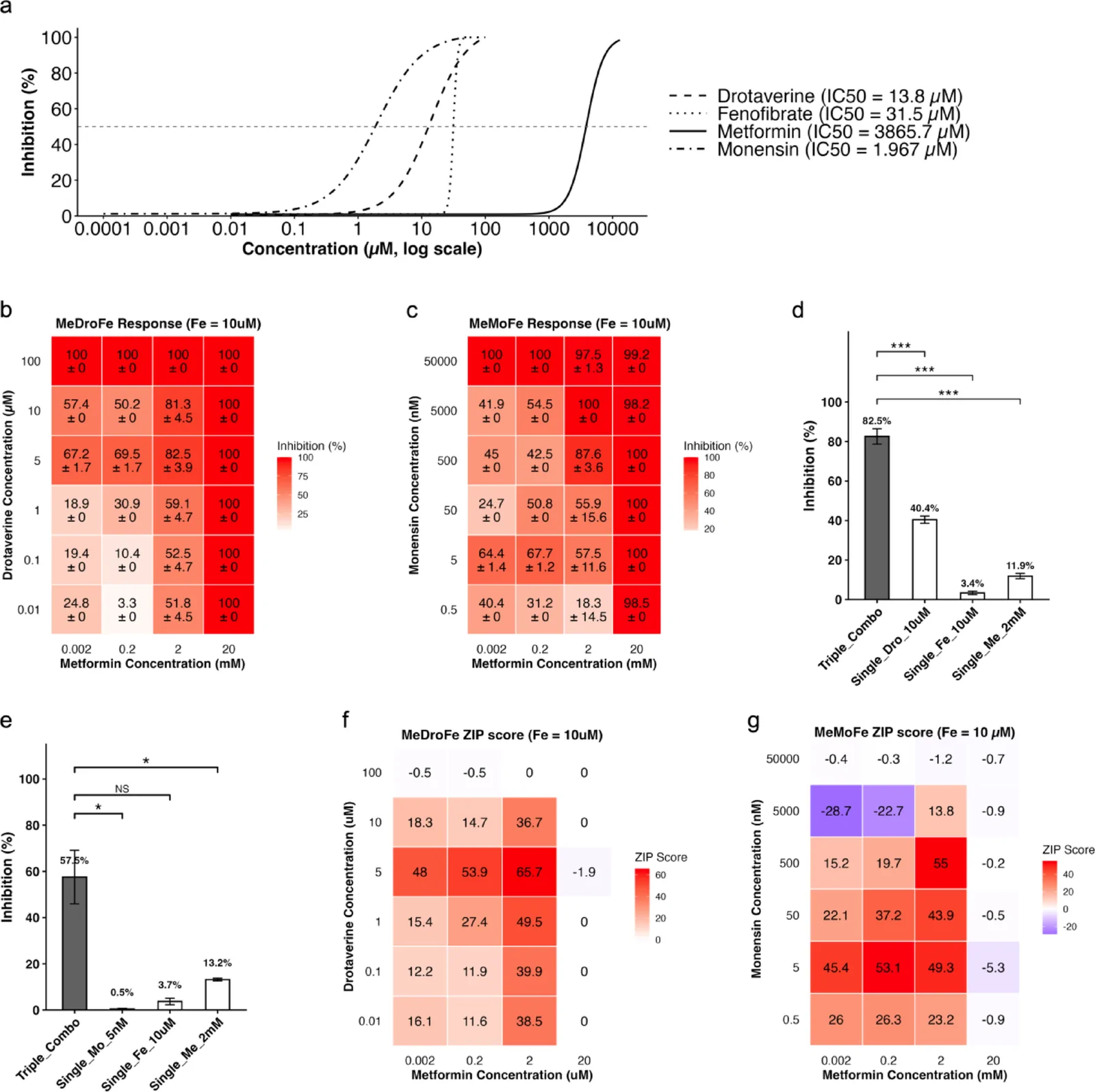
Inhibition of MM1.S cell viability by single-agent and combination therapies. (**a**) IC50 curves of metformin (Me), fenofibrate (Fe), drotaverine (Dro), and monensin (Mo) determined using a four-parameter logistic (4PL) variable-slope dose-response model after 72 h of treatment. (**b**, **c**) Heatmaps showing mean percentage viability inhibition across increasing concentrations of Me paired with (**b**) Dro or (**c**) Mo, in the presence of a constant concentration of Fe (10 µM). Data represent mean ± standard error of the mean (SEM). (**d**, **e**) Comparative inhibition following treatment with single agents or triple-drug combinations: (d) MeDroFe (Me 2 mM, Dro 5 µM, Fe 10 µM) or (**e**) MeMoFe (Me 2 mM, Mo 5 nM, Fe 10 µM). (**f**, **g**) Zero Interaction Potency (ZIP) synergy score heatmaps for 2D concentration matrices of Me paired with (**f**) Dro or (**g**) Mo at a fixed Fe concentration (10 µM). Cell viability values were min – max normalized prior to synergy calculation (Percent Inhibition = 100 - Percent Viability). Synergy scores and matrices were computed using the SynergyFinder package in R. Data represent mean ± SEM. n = 3 technical replicates. ANOVA analysis and Dunnett’s post hoc test: * P < 0.05, *** P < 0.001, NS = not significant.

Statistical analysis was performed with Prism Version 8 (GraphPad, Boston, MA) and R Statistical Software (Version 2026.7.1.147) [16]. Testing for statistical significance was performed with one-way or two-way ANOVA with Dunnett post-hoc analysis for comparing three groups for two or more variables. The normal distribution was assessed using the Shapiro-Wilk test and Q-Q plots. Although the Shapiro-Wilk test and Q-Q plots indicated mild deviations from normality for the viability inhibition data (Fig. 1d and e), group sample sizes were equal and variances were homogeneous. Consequently, a standard ANOVA was performed, as the test remains robust to minor normality violations under these specific conditions. IC_50_ values were calculated using a four-parameter logistic (4PL) variable-slope dose-response in R. The Zero Interaction Potency (ZIP) model was used for 3-drug synergy analysis and was performed using the “SynergyFinder” package in R (version 2026.7.1.147) [16, 17]. The figure legends provide a detailed description of the statistical testing for each dataset. N represents the number of independent technical or biological replicates, each performed at least in technical triplicates. Unless otherwise stated in the figure legends, experiments were performed with a minimum of *n* = 3 technical or biological replicates.

The quantification of gene expression data derived from quantitative polymerase chain reaction (RT-qPCR) experiments was performed by using the 2^−ΔΔCt^ method (*RPL13* was analyzed as reference gene). The normal distribution was assessed using the Shapiro-Wilk test and Q-Q plots. The statistical significance of the comparison between gene expression of hMCLs vs. hOBs was determined by performing a Mann-Whitney U test, given the non-normal distribution of this dataset. Comparison of *SLC2A8* and *DDIT3* expression of three hMCLs was performed with two-way ANOVA with Dunnett post-hoc analysis.

In the absence of other indications, the mean with standard error is indicated. An error probability of *< 0.05* was considered statistically significant.

## Results

### The combination of MCI inhibitors with H^+^-ionophores results in a higher cytotoxicity than a single-drug treatment in hMCLs

We investigated the impact of the mitochondrial complex I (MCI) inhibitors metformin (Me) and fenofibrate (Fe) combined with the H^+^-ionophores drotaverine (Dro) or monensin (Mo) on the cell viability of hMCLs. To determine appropriate treatment doses, we first evaluated each agent individually across a range of concentrations in MM1.S cells to establish their respective IC_50_ values (Fig. 1a).

Anticipating synergistic effects from the triple-combination regimens, our objective was to select single-drug concentrations that exhibited minimal individual cytotoxicity. Based on the calculate IC_50_ values (Fig. 1a) for metformin (3.9 mM), fenofibrate (31.5 µM), drotaverine (13.8 µM), and monensin (1967 nM), we sought lower concentrations for use in the combination studies. Because fenofibrate displayed a steep dose-response curve with marked cytotoxicity at higher doses, we fixed its concentration at 10 µM based on our prior experience with WT-drug combination models. Using this fixed fenofibrate baseline, we next evaluated concentration matrices with the H^+^-ionophores drotaverine and monensin (Fig. 1b–c). When combined with 2 mM metformin, drotaverine exhibited potent cytotoxicity starting at 5 µM reaching a maximum inhibition of 82.5% (Fig. 1b). Conversely, monensin induced cytotoxicity even at low doses when combined with Me and Fe; therefore, we selected a low dose of 5 nM for further investigation (Fig. 1c).

To determine whether the triple combinations provided superior efficacy over monotherapies at identical doses, we evaluated the triple-drug combinations MeDroFe (metformin, drotaverine, and fenofibrate) and MeMoFe (metformin, monensin, and fenofibrate) (Fig. 1d – e). The MeDroFe combination (2 mM metformin, 5 µM drotaverine, and 10 µM fenofibrate) achieved 82.55% ± 3.92% inhibition, which was significantly greater than single-agent cytotoxicity induced by 2 mM metformin alone (11.89% ± 1.35%, *P* < 0.001), 10 µM fenofibrate alone (3.37% ± 0.87%, *P* < 0.001) or 5 µM drotaverine alone (40.43% ± 1.82%, *P* < 0.001; Fig. 1d). Similarly, the MeMoFe triple combination (2 mM metformin, 5 nM monensin, and 10 µM fenofibrate) substantially reduced cell viability (57.5% ± 11.6%) compared to monensin or metformin alone (0.47% ± 0.26%, monensin *P* < 0.05 and metformin *P* < 0.05) (Fig. 1e).

To validate synergistic interaction, we performed Zero Interaction Potency (ZIP) synergy analyses across all three-drug combinations (Supplementary Fig. 1). The MeDroFe combination (2 mM Me, 5 µM Dro, 10 µM Fe) produced the highest ZIP synergy score (65.7) among all tested drotaverine/metformin pairs, indicating strong synergy (Fig. 1f). For MeMoFe, while the maximum ZIP score (55.0) occurred at 500 nM monensin and 2 mM metformin, the 5 nM monensin and 2 mM metformin pair demonstrated also a robust ZIP score of 49.3. Furthermore, this lower monensin concentration maintained strong synergistic interactions across reduced metformin doses (0.2 mM Me ZIP score: 53.1; 0.02 mM Me ZIP score: 45.4) (Fig. 1g). We further evaluated varying fenofibrate concentrations within the triple combination; however, concentrations both lower and higher than 10 µM yielded lower ZIP synergy scores (Supplementary Fig. 1). Given these pronounced synergistic profiles and the significant enhancement in cytotoxicity over monotherapies, the combination of 2 mM metformin, 5 nM monensin, and 10 µM fenofibrate was selected for subsequent experiments.

### WT drug combinations induce pH-dependent cytotoxicity and intracellular acidification in hMCLs while simultaneously inhibiting cell migration

**Fig. 2:**
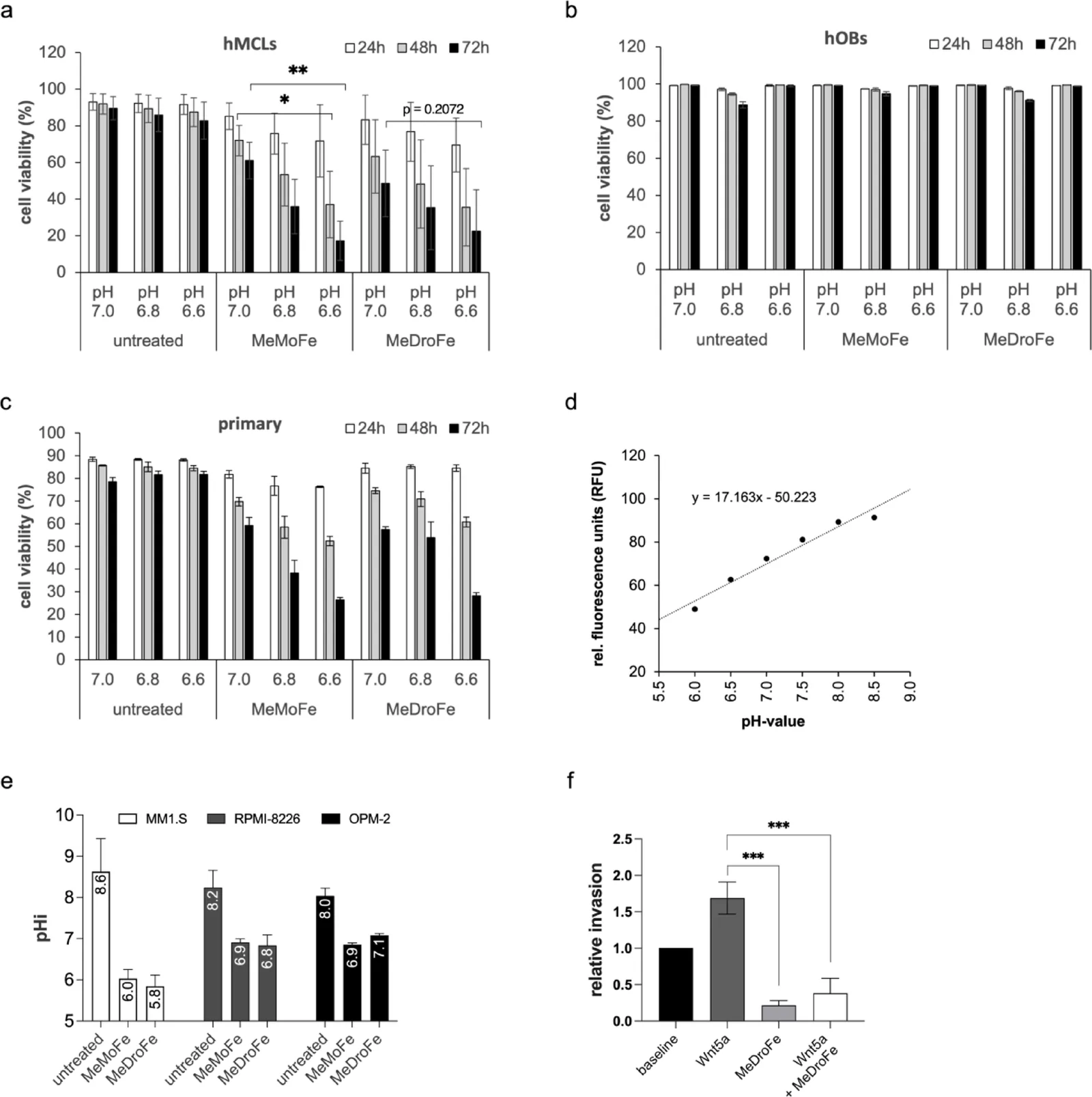
Impairment of hMCLs, hOBs and primary MM cells following WT drug treatment and intracellular acidification of hMCLs after treatment. (**a**-**c**) Cell viability of (**a**) hMCLs, (**b**) human osteoblasts (hOBs) and (**c**) primary MM cells treated with MeMoFe or MeDroFe in cell culture medium with a pH-value of 7.0, 6.8 or 6.6. for 24, 48, and 72 h. Untreated cells served as controls. (**d**) Exemplary standard curve showing the measurement of intracellular pH (pHi) values in MM1.S cells cultured in medium with the indicated pH values and the addition of the ionophore nigericin (10 µM). These standard curves were used to calculate the pHi-values in the experimental samples. (**e**) pHi of MM1.S, RPMI-8226 and OPM-2 cell lines was measured following a 72-hour treatment with MeMoFe, MeDroFe or without treatment. (**f**) Relative invasiveness of two hMCLs (RPMI-8226 and MM1.S) was measured after 24 h of treatment with MeDroFe, rWnt5a (250 ng/ml), or both. Data is presented as means ± standard deviation (SD). n = 3 biological replicates for (**a**), n = 3 technical replicates for (**b**) and (**c**) and n = 4 technical replicates for (**f**). Statistical analysis was performed by comparing multiple groups with two-way ANOVA and Dunnett’s post-hoc analysis: * P < 0.05, ** P < 0.01, *** P < 0.001

Initial single- versus triple-drug combination testing was performed in a cell culture medium at a physiological pH of 7.2–7.4, which approximates the pH of benign bone marrow. However, in vivo measurements have revealed that the extracellular pH in multiple myeloma ranges between 6.7 and 6.8 [12], a more acidic microenvironment than benign bone marrow. Therefore, we evaluated the efficacy of WT drug combination therapies in acidic cell culture media to mimic the MM tumor microenvironment. The viability of untreated hMCLs was largely unaffected by acidic conditions (82.80% ± 10.17% at pH 6.6 after 72 h), but treatment with MeMoFe or MeDroFe both impaired cellular viability under physiological and acidic pH conditions (Fig. 2a). Both drug combinations induced the strongest inhibition of viability after 72 h at pH 6.6 (Fig. 2a). Specifically, the MeMoFe combination reduced viability to 17.27% ± 10.76% at pH 6.6 after 72 h, which was significantly lower than the viability observed at pH 7.0 (61.04%, *P* < 0.01; Fig. 2a). MeDroFe exhibited greater inhibition than MeMoFe at pH 7.0 after 72 h; however, its inhibitory effect at pH 6.6 was slightly lower, resulting in residual viability of 22.52% ± 22.66% (Fig. 2a).

To evaluate whether the selected drug concentrations might adversely affect the viability of non-neoplastic cells, we tested the treatment on primary human osteoblasts (hOBs). The cellular viability of hOBs treated with MeMoFe and MeDroFe was not impaired compared to the untreated control group, regardless of the pH-value (Fig. 2b).

We further tested our hypothesis of acidity-enhanced cytotoxicity in primary MM cells, which exhibited a response to the treatment similar to that of hMCLs (Fig. 2c). The most pronounced effect was again observed after 72 h of treatment, with greater efficacy at lower pH levels. Both MeMoFe and MeDroFe achieved maximum suppression of viability at pH 6.6 (26.45% ± 1.10% and 28.25% ± 1.45%, respectively), underscoring the effect observed in hMCLs.

To validate the hypothesized central mechanism underlying the impairment of viability by WT treatment, namely intracellular hyper-acidification, we measured the intracellular pH (pHi) of hMCLs treated with MeDroFe and MeMoFe (exemplary standard curve of pHi measurement in Fig. 2d).

Our analysis revealed a markedly lower pHi in WT drug-treated cells compared to untreated controls. This effect was most pronounced in MM1.S cells with a drop in pHi from 8.6 (untreated) to 5.8 (MeDroFe). The decreases in pHi for RPMI-8226 and OPM-2 cells were comparable to those in MM1.S, declining from 8.2 to 6.8 (RPMI-8226) and from 8.0 to 6.9 (OPM-2) (Fig. 2e). These findings demonstrate intracellular hyper-acidification as a consequence of WT drug treatment in hMCLs.

To determine if WT treatment affects capabilities beyond cell viability, we investigated its impact on the invasiveness of hMCLs using a transwell migration assay that simulates basement membrane invasion. As a positive control, we used recombinant Wnt5a (rWnt5a), a non-canonical Wnt ligand that is known to enhance hematologic cancer cell motility [18, 19]. Accordingly, treatment with rWnt5a increased the invasive capacity of hMCLs by more than 1.5-fold compared to baseline, confirming its utility as a positive control (Fig. 2f). Compared to this positive control, treatment with the WT drug combination MeDroFe significantly suppressed cellular invasion to 0.2-fold relative to baseline (*P* < 0.001, Fig. 2f). Notably, WT drug treatment also suppressed Wnt5a-induced invasion to levels below baseline migration rates, even when rWnt5a was applied prior to treatment (Fig. 2f), suggesting a suppressive effect of WT therapy on the migratory and invasive capacity of hMCLs. The observed reduction in migration may be due, at least in part, to decreased cell viability. However, cell viability remains above 80% after 24 h (Fig. 2a), which is the time point at which the migration analysis was performed. This suggests a direct effect of the applied WT drugs.

In conclusion, these findings suggest that the acidic microenvironment characteristic of the MM bone marrow niche enhances the cytotoxic efficacy of WT drug combinations (MeMoFe and MeDroFe) in both, hMCLs and primary MM cells, while sparing non-neoplastic osteoblasts. This cytotoxicity is chaperoned by intracellular hyper-acidification. Furthermore, treatment with WT effectively abrogates both basal and Wnt5a-induced cell invasion, suggesting a potential dual role in impairing MM cell survival and migration capacity.

### WT-drug combination therapy induces Caspase-dependent apoptosis of hMCLs

**Fig. 3:**
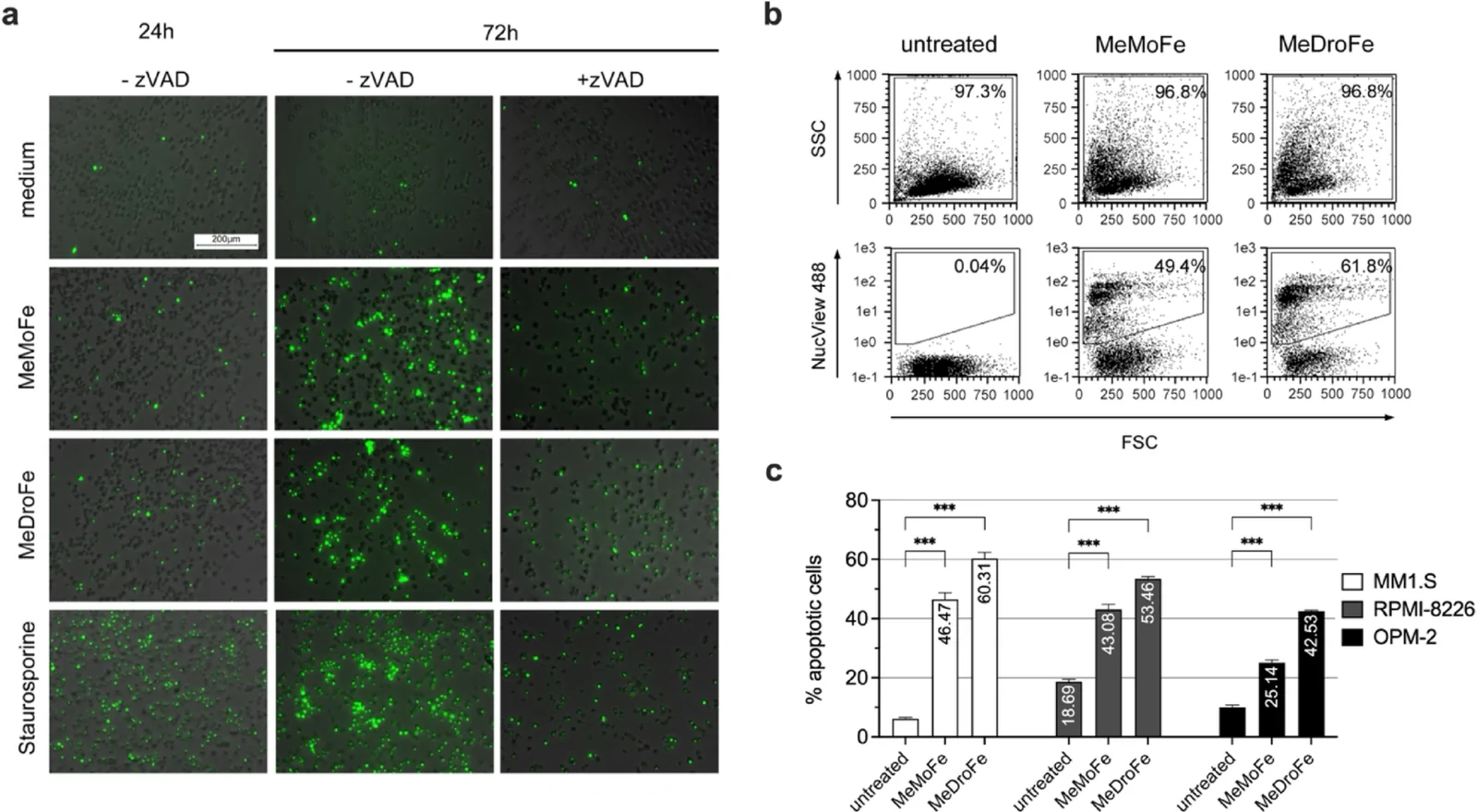
Detection of apoptosis in hMCLs following WT drug treatment. (**a**) Induction of apoptosis was detected in MM1.S cells using the fluorescent substrate NucView 488 that detects active caspase-3/7, a hallmark of apoptosis. Cells were treated with MeMoFe or MeDroFe with or without addition of the pan-caspase inhibitor zVAD. The protein kinase C inhibitor and known apoptosis inducer Staurosporine served as a positive control. Images of green fluorescence were captured at 200x magnification and merged with the corresponding bright-field images. (**b**) Representative flow cytometry plots illustrating the gating strategy: After exclusion of the debris with a FSC (forward scatter) / SSC (side scatter) gate (upper row), apoptotic cells were gated based on the NucView 488 fluorescence intensity (bottom row). The percentages of gated cells are indicated. (**c**) Quantification of apoptotic MM1.S, RPMI-8226 and OPM-2 cells by flow cytometry after 72 h of treatment with MeMoFe or MeDroFe. Data represent mean ± SD. n = 3 technical replicates for (**c**). Statistical analysis was performed by comparing treated groups to the untreated control group with two-way ANOVA and Dunnett’s post-hoc analysis: *** P < 0.001

A potential induction of apoptosis by the WT-drugs was investigated through the analysis of caspase-3/7 activity in MM1.S cells after treatment with MeMoFe or MeDroFe. Apoptotic cells were identified by fluorescence microscopy and quantified by flow cytometry using a caspase-3/7-dependent fluorescent dye. This analysis revealed strong apoptosis signals in MM1.S cells exposed to WT-drug treatment, with an increasing number of apoptotic cells observed with longer treatment durations (Fig. 3a). The specificity of the caspase-3/7 signal was validated using the pan-caspase inhibitor zVAD (Fig. 3a). In the presence of zVAD, the caspase-3/7-dependent fluorescent signal was markedly reduced, resembling the negative control, thereby confirming the specificity of the caspase-3/7 staining (Fig. 3a). Quantification of apoptotic cells in all three hMCLs revealed a significant increase following combination therapy, as opposed to the untreated control groups (*P* < 0.001, Fig. 3c). Notably, MeDroFe produced a stronger apoptotic response than MeMoFe in all three hMCLs (Fig. 3c).

Consistent with the pHi analysis, whereby MM1.S cells achieved the greatest drop in pHi to 5.8 after MeDroFe, MM1.S cells exhibited the highest percentage of apoptotic cells with 60.3% ± 2.08% after 72 h, suggesting that this cell line was the most vulnerable to the WT-treatment (Fig. 3c). To conclude, WT-drug treatment induced caspase-dependent apoptosis in all tested hMCLs. However, cell line-dependent differences were observed, with MM1.S exhibiting the highest apoptosis rate among all the cell lines examined.

### The response to WT-treatment results in metabolic dysregulation, leading to ATP depletion and mitochondrial depolarization

**Fig. 4:**
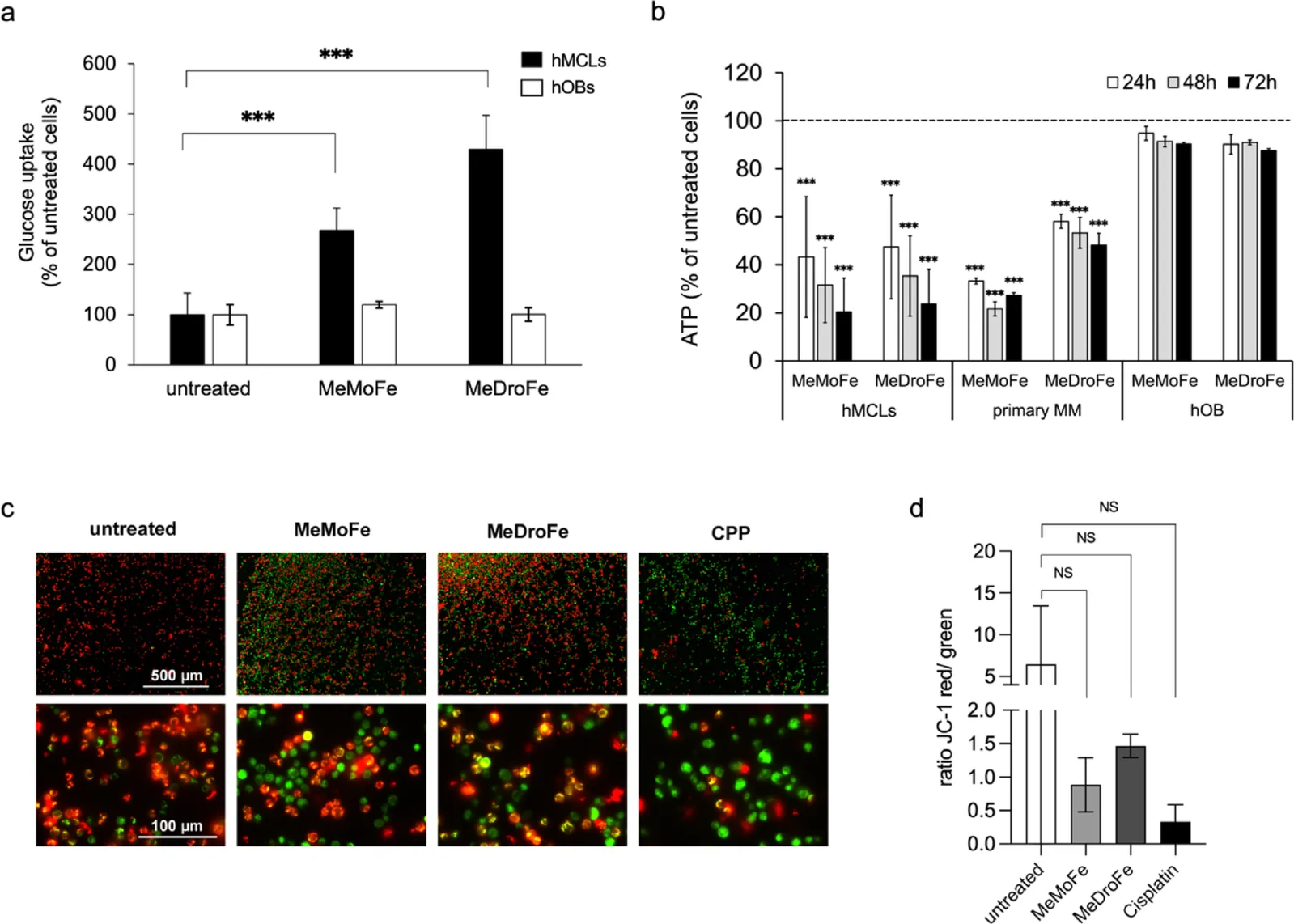
MeMoFe and MeDroFe induce compensatory glucose uptake, ATP depletion and mitochondrial depolarization in hMCLs. (**a**) Quantification of cellular glucose uptake in hMCLs and hOBs following 24 h treatment with MeMoFe, MeDroFe, or no treatment; expressed as a percentage relative to untreated control. (**b**) Quantification of ATP levels in hMCLs, primary MMs and hOBs. Cells were treated with MeMoFe or MeDroFe for the indicated time before ATP levels were analyzed and expressed as percentage relative to untreated control. (**c**) Representative fluorescence microscopy images of MM1.S cells stained with JC-1 following 24 h treatment with MeMoFe / MeDroFe, cisplatin (CPP) as positive control, or no treatment. Red fluorescence represents intact mitochondrial membrane potential, while green fluorescence indicates mitochondrial depolarization (JC-1 monomers). Scale bars: 500 μm and 100 μm. (**d**) Quantification of JC-1 fluorescence signal in MM1.S cells as ratio red fluorescence to green fluorescence following treatment with MeMoFe, MeDroFe or cisplatin for 24 h. All data presented as mean ± SD. n = 3 biological replicates for hMCLs in (**a**) and (**b**), n = 3 technical replicates for primary MM and hOBs, n = 3 technical replicates for (**d**). Statistical analysis was performed by comparing treated groups to the untreated control group with two-way ANOVA and Dunnett’s post-hoc analysis (**a**) and (**c**) or Tukey’s post hoc analysis (b): *** P < 0.001, NS = not significant

Building on the observed effects of the WT drug treatment, we sought to elucidate the mechanism driving apoptosis. We hypothesized that metformin disrupts the mitochondrial electron transport chain via MCI inhibition, causing a decline in ATP production and a compensatory increase in glycolysis [14]. To test this, we first evaluated glucose uptake in hMCLs and hOBs following treatment. Compared to untreated controls, MeMoFe increased glucose uptake in hMCLs more than 2.5-fold (268.22% ± 44.30%, *P* < 0.001; Fig. 4a). This effect was even more pronounced with MeDroFe, which induced a greater than 4-fold increase in glucose uptake (429.00% ± 68.05, *P* < 0.001; Fig. 4a). In contrast, the same treatments in non-neoplastic hOBs elicited only a modest response with MeMoFe (119.83% ± 6.78%), and no detectable change with MeDroFe (100.62% ± 13.51%; Fig. 4a), indicating that this elevated glucose uptake selectively occurs in hMCLs.

We further hypothesized that the combination of MCI inhibitors with H^+^-ionophores would accelerate glucose consumption through the utilization of proton pumps to counteract ionophore-induced intracellular acidification, thereby exacerbating ATP depletion [14, 15]. Consistent with this model, intracellular ATP levels steadily declined over time in both hMCLs and primary MM cells following MeMoFe and MeDroFe exposure (Fig. 4b). The most pronounced reduction occurred after 72 h in hMCLs, reaching 20.46% ± 14.01% (MeMoFe) and 23.72% ± 14.47% (MeDroFe) of untreated control levels (*P* < 0.001, Fig. 4b). ATP depletion was slightly less pronounced in primary MM cells, with remaining ATP levels of 27.26% ± 1.11% under MeMoFe and 48.23 ± 4.89% under MeDroFe, compared to untreated controls (*P* < 0.001, Fig. 4b). It should be noted that the observed reduction of ATP levels may partly be a consequence of decreased cell viability in response to WT treatment. However, the main decline in ATP concentration occurs during the first 24 h, when cell viability is still high, suggesting a direct impact of the WT drugs on ATP depletion (Fig. 4b). Importantly, hOBs exhibited minimal ATP loss under both MeMoFe (90.36% ± 0.67%, NS) and MeDroFe (87.53% ± 0.86%, NS) after 72 h compared to untreated controls (Fig. 4b).

Given the disruption of mitochondrial function downstream of MCI inhibition, we evaluated whether this mechanism causes mitochondrial membrane depolarization contributing to WT-drug cytotoxicity. The mitochondrial membrane potential of treated MM1.S cells was monitored using JC-1 staining. This dye exhibits red fluorescence in the intact mitochondria of healthy cells but shifts to green fluorescence upon membrane depolarization. Cisplatin was used as a positive control, confirming the transition from red to green fluorescence during mitochondrial membrane depolarization (Fig. 4c). Representative fluorescence micrographs of MM1.S cells demonstrated a marked increase in green signal following WT drug treatment, indicating loss of mitochondrial membrane potential (Fig. 4c). Quantification of the red / green fluorescence ratio confirmed a shift toward depolarization as shown by a drop of these ratios from 6.5 in untreated cells to 0.9, 1.5 and 0.3 in cells treated with MeMoFe, MeDroFe and cisplatin, respectively (Fig. 4d).

In summary, our data support the proposed mechanism of WT drug induced cell death. The underlying cause appears to be a disruption of energy metabolism, which triggers a compensatory glycolytic cascade. The observed increase in glucose uptake is presumably the cause of intracellular acidification, which ultimately leads to ATP depletion, loss of the mitochondrial membrane potential, and apoptosis. However, further studies are needed to confirm whether this sequence actually occurs in the order described. Notably, the observed reactions are exclusive to neoplastic cells, whereas non-neoplastic hOBs tolerate WT drug treatment well.

### *SLC2A8* and *DDIT3* transcriptional induction in MM1.S compared to hOBs and other hMCLs

Given that MM1.S cells exhibited the most pronounced apoptotic response and intracellular acidification under WT drug treatment, we further explored whether baseline differences in the expression of glucose transporters might correlate with the observed heterogeneity in treatment sensitivity. Therefore, we performed RT-qPCR profiling in the three hMCLs and non-neoplastic hOBs.

**Fig. 5:**
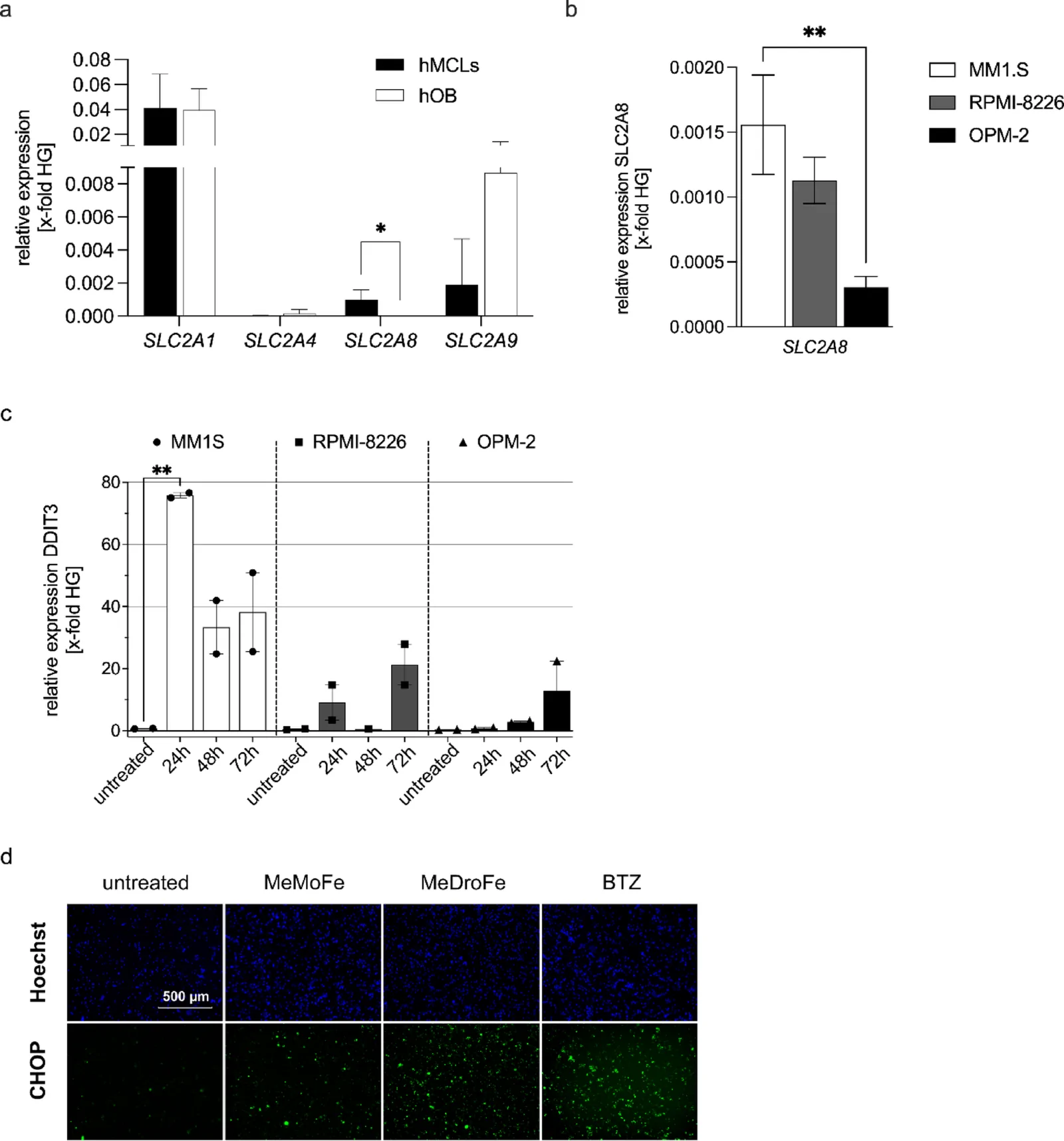
Expression of GLUT protein related genes and DDIT3 in hMCLs. (**a**) Relative expression of SLC2A1 (GLUT1), SLC2A4 (GLUT4), SLC2A8 (GLUT8) and SLC2A9 (GLUT9) mRNA encoded by SLC2A-family genes of untreated hMCLs and hOBs quantified by RT-qPCR. Data represent mean ± SD. (**b**) Expression of SLC2A8 in MM1.S, RPMI-8226 and OPM-2 cell lines quantified by RT-qPCR. Data represent mean ± SD. (**c**) Relative expression of cellular stress associated gene DDIT3 in MM1.S, RPMI-8226 and OPM-2 cell lines after 24 h, 48 h and 72 h of treatment with MeDroFe or without treatment. Relative quantification is displayed and normalized to the housekeeping gene (HG) RPL13. Data represent median ± range. (**d**) Detection of CHOP protein by Immunocytochemistry. Representative images of RPMI-8226 cells are shown after treatment with MeMoFe, MeDroFe or 50 nM Bortezomib (BTZ) for 24 h. Green fluorescence represents CHOP and blue fluorescence represents a Hoechst 33,342 nuclear counterstain. n = 3 biological replicates for hMCLs and n = 3 technical replicates for hOBs in (**a**), n = 3 technical replicates in (**b**), n = 2 technical replicates in (**c**). Statistical analysis was performed by Mann-Whitney-U-test in (**a**) and two-way ANOVA and Dunnett’s post-hoc analysis (**b**) and (**c**): * P < 0.05, ** P < 0.01.

Glucose uptake in human cells is mediated by fourteen solute carrier family 2 A (*SLC2A*) gene products encoding glucose transporters (GLUTs) [20]. Based on prior evidence documenting their expression in human MM cells [21], we analyzed a targeted panel comprising *SLC2A1*, *SLC2A4*, *SLC2A8*, and *SLC2A9*. Expression of *SLC2A4* and *SLC2A9* was negligible across all tested hMCLs (Fig. 5a). In contrast, *SLC2A1* (encoding GLUT1) was the most abundant transcript among the evaluated *SLC2A* genes in both hMCLs and hOBs (Fig. 5a). The most notable distinction occurred in *SLC2A8* (encoding GLUT8), which exhibited significantly higher baseline expression in hMCLs than in hOBs (*P* < 0.05; Fig. 5a). Specifically, MM1.S cells, which displayed the highest apoptotic susceptibility to WT drug treatment, expressed 5-fold higher *SLC2A8* transcript levels than OPM-2 cells (Fig. 5b). RPMI-8226 cells, which demonstrated intermediate sensitivity, also displayed elevated *SLC2A8* expression relative to OPM-2 cells (Fig. 5b). While these expression profiles align with the differential sensitivity across cell lines, further analyses (e.g. direct flux analysis) will be required to confirm whether this transcriptional variation translates to altered glucose metabolism.

To further characterize downstream transcriptional stress pathways induced by WT drug exposure, we examined mRNA expression of DNA Damage Inducible Transcript 3 (*DDIT3*, encoding CHOP), a recognized transcriptional component of the cellular stress cascade and endoplasmic reticulum (ER) stress-mediated apoptosis [22]. Standard frontline MM therapies such as proteasome inhibitors trigger lethal proteotoxic and ER stress by impairing misfolded protein clearance. Given the proposed involvement of GLUT8 in supporting high-rate immunoglobulin processing in plasma cells [21], we investigated whether WT drug exposure alters *DDIT3* transcription. Treatment induced high expression of *DDIT3* mRNA in MM1.S cells, peaking at 24 h with a 75.9-fold induction compared to untreated controls, and persisting at more than 30-fold and 38-fold elevations at 48 h and 72 h, respectively (Fig. 5c). In RPMI-8226 and OPM-2 cells, *DDIT3* transcript levels trended upward, reaching their highest levels after 72 h of exposure (Fig. 5c). In addition, a nuclear accumulation of the DDIT3 encoded CHOP protein was observed after 24 h of treatment with MeMoFe, MeDroFe and bortezomib. The proteasome inhibitor bortezomib, used in the treatment of multiple myeloma, is a known inducer of ER stress [23] and served as a positive control. Representative fluorescence images of the RPMI-8226 cell line are shown in Fig. 5 (Fig. 5d).

In conclusion, these data suggest differential *SLC2A8* expression patterns across hMCLs and identify an early induction of *DDIT3* in MM1.S cells compared to a delayed trend in other hMCLs following WT drug treatment. Additionally, we noted a qualitative increase in CHOP protein expression in hMCLs after treatment with MeMoFe and MeDroFe. As this accumulation was not quantified and our overall findings remain descriptive, further functional studies are necessary to elucidate whether a mechanistic link exists between glucose transporter expression, cellular stress signaling, and treatment sensitivity.

## Discussion

In this study, we targeted the most well-known metabolic alteration in cancer cell energy metabolism, known as the Warburg effect. Specifically, we sought to extend the application of the “Warburg Trap” (WT) drug combination, a concept our group previously established and evaluated in osteosarcoma models [14]. This combination consists of the clinically approved MCI inhibitor metformin in combination with a H^+^-ionophore (e.g. monensin or drotaverine) [14, 15].

Metformin has well-documented anti-cancer properties and has been associated with improved overall survival in patients with MM [24]. Extensive literature demonstrates that metformin can inhibit MM cell proliferation, induce apoptosis, and sensitize tumor cells to other therapies by modulating pathways such as IGF-1R/PI3K/AKT/mTOR and DNA damage repair [25–29]. However, in our current study, metformin monotherapy exhibited relatively limited cytotoxic effects on hMCLs at the same concentration we used it in our combination therapy. This apparent discrepancy aligns with the broader cancer metabolism literature, which indicates that metformin typically requires high, often supraphysiological concentrations to achieve single-agent efficacy in vitro [29, 30]. Studies investigating metformin monotherapy in vitro commonly used more than 5-fold the dosage used in our combination approach [26, 29, 30].

Clinically, achieving these high doses is hindered by metformin’s low oral bioavailability (40–60%), highly hydrophilic nature, and lack of plasma protein binding, which collectively limit its uptake by tumor tissue. Furthermore, high-dose administration increases the risk of severe adverse effects, such as lactic acidosis [31]. Consequently, several studies have explored combining metformin with other agents, such as fingolimod, ritonavir, or established anti-MM drugs like bortezomib, to enhance its cytotoxicity at lower doses [28, 32, 33]. However, even in these combination strategies, the required metformin concentrations remained 2- to 10-fold higher than those utilized in our approach [25, 28, 33].

Our WT approach builds upon this rationale by combining MCI inhibitors (metformin or fenofibrate) with H^+^-ionophores to forcefully target cancer cell energy metabolism [14, 15]. As we previously described in our osteosarcoma models [14], the aim of inhibiting the mitochondrial complex I with metformin or fenofibrate was to force the tumor cells to generate energy exclusively through glycolysis. The ability of the cells to counteract the resulting intracellular acidification should then be prevented by means of H^+^-ionophores like monensin or drotaverine. Here, we successfully applied this established approach to MM. In accordance with prior WT studies, combination treatment induced intracellular hyper-acidification and apoptosis. Supporting our hypothesis, these effects were accompanied by an increased glucose uptake, decreased ATP levels and mitochondrial depolarisation. Notably, the cytotoxic effects of the tested WT drug combinations were observed in both, hMCLs and primary MM cells, but not in hOBs, which were used as non-neoplastic control cells. It is also important to note that the drugs act synergistically, resulting in significantly lower concentrations of the individual drugs in the combinations compared to single-drug treatments.

Because WT efficacy relies heavily on glucose uptake, we also investigated GLUT gene expression and noted a strong upregulation of GLUT8 in the most sensitive cell line. This suggests a possible association with glucose uptake, although these conclusions remain heavily limited without direct metabolic flux analyses. Interestingly, this finding is consistent with previous reports highlighting the importance of GLUT8 in MM viability [7, 21, 34]. Furthermore, GLUT8 is predominantly located intracellularly and associated with the exocytic compartment, suggesting a potential role in the production of immunoglobulins within the ER of MM cells [21]. To evaluate ER stress in MM cells following WT treatment, we investigated the induction of DDIT3 (encoding the transcription factor CHOP), a well-established ER stress marker [22, 35]. Consistent with our previous observations in other tumor entities, treatment induced DDIT3/CHOP gene and protein expression, suggesting the induction of ER stress in response to WT drug treatment [15, 36]. However, we acknowledge the limitations of our current study, as these descriptive data do not provide sufficient mechanistic evidence to definitively link DDIT3/CHOP upregulation to ER stress-mediated responses following WT treatment.

To enhance the translational feasibility of our approach, we focused on combinations utilizing FDA-approved drugs, substituting the veterinary antibiotic monensin with drotaverine. Drotaverine, approved in numerous countries as a smooth muscle relaxant [36], acts as a phosphodiesterase inhibitor that indirectly suppresses TCA cycle flux. We also tested fenofibrate, which offers a dual mechanism by inhibiting both MCI [37]and MCT4 (Monocarboxylate transporter 4) [38], thereby hypothetically amplifying intracellular acidification. The synergistic nature of these combinations allows for significantly lower drug concentrations, minimizing the risk of adverse effects associated with high-dose monotherapies and distinguishing our approach from other metabolic targeting strategies. While previous metabolic approaches have focused on targeting single key enzymes or depriving MM cells of nutrients [10, 32, 39], our approach actively exploits the cancer cell’s reliance on aerobic glycolysis to create a devastating, auto-enhancing cycle. Many targeted metabolic inhibitors (e.g., experimental LDHA or ASCT2 inhibitors) lack established safety profiles or FDA approval. In contrast, the WT approach focuses on repurposing a combination of widely used, clinically approved drugs (e.g., metformin, fenofibrate, and drotaverine), potentially offering a faster and safer route to clinical translation.

While repurposing clinically established drugs offers a strong therapeutic rationale, we recognize the highly complex and multifaceted nature of the malignant bone marrow microenvironment [39–43]. A primary limitation of our study lies in its in vitro proof-of-concept design; therefore, conclusions regarding broad clinical applicability are premature. Regarding safety and selectivity, we observed that the WT combination spared human osteoblasts, aligning with our previous studies [14]. However, evaluating a single non-malignant cell type provides only a preliminary indication of tolerability. Further evaluations incorporating a broader panel of non-malignant, bone marrow-relevant control cells are required.

Before this WT strategy can be considered for translation, future preclinical studies must prioritize robust in vivo efficacy validation, comprehensive pharmacokinetic and pharmacodynamic (PK/PD) profiling and evaluations of these regimens in combination with current standard-of-care anti-myeloma therapies.

We envision our approach as a potential add-on or maintenance therapy. New therapy options, such as CAR T-cell and bispecific antibodies, have increased the average multiple myeloma survival rate to over 10 years [4, 5, 44, 45]. However, these therapies can also cause severe adverse side effects, which underscores the need for supportive therapies that are effective, have minimal side effects, and can suppress MM cell growth over a prolonged period of time.

## Supplementary Information

Below is the link to the electronic supplementary material.

Supplementary Material 1

## Data Availability

The datasets used and/or analysed data during the current study are available from the corresponding author on reasonable request.

## Abbreviations

ATP: Adenosine triphosphate
ASCT2: Alanine, serine, cysteine transporter 2
BSA: Bovine serum albumin
BTZ: Bortezomib
DMSO: Dimethyl sulfoxide
FBS: Fetal bovine serum
FDG-PET: Fluorodeoxyglucose positron emission tomography
GLUT: Glucose transporter
HBSS: Hank’s balanced salt solution
HK2: Hexokinase II
hMCLs: Human myeloma cell lines
hOBs: Human osteoblasts
LDHA: Lactate dehydrogenase A
MCI: Mitochondrial complex I
MCT4: Monocarboxylate transporter 4
MM: Multiple myeloma
PBS: Phosphate buffered saline
PI: Propidium iodide
PKM2: Pyruvate kinase M2
RT-qPCR: Reverse transcription quantitative polymerase chain reaction
SD: Standard deviation
SEM: Standard error of the mean
SLC2: Solute carrier family 2
TCA: Tricarboxylic acid cycle
WT: Warburg Trap
ZIP: Zero interaction potency
